# Sharp Changes in Muscle Tone in Humans Under Simulated Microgravity

**DOI:** 10.3389/fphys.2021.661922

**Published:** 2021-05-06

**Authors:** Liubov E. Amirova, Anastasija Plehuna, Ilya V. Rukavishnikov, Alina A. Saveko, Aleko Peipsi, Elena S. Tomilovskaya

**Affiliations:** ^1^Laboratory of Gravitational Physiology of the Sensorimotor System, Institute of Biomedical Problems, Russian Academy of Sciences, Moscow, Russia; ^2^King’s College London, Centre of Human & Applied Physiological Sciences, London, United Kingdom; ^3^Department of Medical Support for Spaceflight, Institute of Biomedical Problem of Russian Academy of Science, Moscow, Russia; ^4^Myoton AS, Tallinn, Estonia

**Keywords:** muscle tone, atony, MyotonPRO, Dry Immersion, microgravity, space flight

## Abstract

A decrease in muscle tone induced by space flight requires a standardized assessment of changes to control the state of the neuromuscular system. This study is a step toward the development of a unified protocol, aimed at determining the initial effect of the presence or withdrawal of support on muscle tone, the effects of a 2-h supportlessness in Dry Immersion (DI) experiments, and the changes in muscle tone depending on the site of measurement. To perform measurements of changes in muscle tone, we used a MyotonPRO device. The list of muscles that we assessed includes: trunk – mm. deltoideus posterior, trapezius, erector spinae; leg – mm. biceps femoris, rectus femoris, tibialis anterior, soleus, gastrocnemius; foot – m. flexor digitorum brevis, tendo Achillis, aponeurosis plantaris. The study involved 12 healthy volunteers (6 men, 6 women) without musculoskeletal disorders and aged 32.8 ± 1.6 years. At the start of DI, there was a significant decrease in muscle tone of the following muscles: mm. tibialis anterior (−10.9%), soleus (−9.6%), erector spinae (−14.4%), and the tendo Achillis (−15.3%). The decrease continued to intensify over the next 2 h. In contrast, the gastrocnemius muscle demonstrated an increase in muscle tone (+7.5%) 2 h after the start of DI compared to the immediate in-bath baseline. Muscle tone values were found to be site-dependent and varied in different projections of mm. erector spinae and soleus. In previous experiments, we observed a high sensitivity of the myotonometry technique, which was confirmed in this study. To make it possible to compare data from different studies, a standardized protocol for measuring muscle tone for general use in gravitational physiology needs to be developed.

## Introduction

Since the creation of the MyotonPRO device, the myotonometry method has become widespread in sports (Kisilewicz et al., 2018) and medicine (Ferreira-Sánchez et al., 2020). Using this method, a relatively large number of studies performed under real and simulated microgravity conditions has been carried out (Lee et al., 2014; Schneider et al., 2015; Treffel et al., 2016; de Abreu et al., 2017; Rukavishnikov et al., 2017; Schoenrock et al., 2018). The Dry Immersion (DI) model is used less often than the bed rest model, but it reproduces neuromuscular deconditioning quite accurately (Navasiolava et al., 2011; Watenpaugh, 2016; Tomilovskaya et al., 2019; Pandiarajan and Hargens, 2020). All studies, even with short exposures such as parabolic flight or several hours of DI, have shown a decrease in muscle tone.

Despite many experiments carried out in real (Gevlich et al., 1983; Kozlovskaya et al., 1990; LeBlanc et al., 2000; Trappe et al., 2009) and simulated weightlessness (Miller et al., 2012), changes in muscle tone induced by hypogravity conditions remain insufficiently investigated. A small number of subjects, different methods of assessment, and the lack of a standard protocol make it difficult to determine quantitative changes in the tone.

There are many approaches to assessing muscle tone or stiffness. In clinical practice, experienced clinicians assess muscle spasticity or atony by means of neurological examination (Martin and Palmer, 2013). Methods for assessing the longitudinal stiffness of muscles are based on the principle of ballistic movement of a limb with a sharp elimination of the force acting on it (Whelan et al., 2018); they are used both in medicine and in sports. Among the methods that can be used to assess the tonic properties of muscles, there are electromyography (Walsh, 1992; Pisano et al., 1996; Zhang et al., 2019) and laser dopplerography. Quite often, the muscle structure is examined using ultrasound to assess the tone or stiffness (Feng et al., 2018; Katsumata et al., 2019). However, most of these methods assess muscle tone by measuring tissue resistance to mechanical pressure exerted by a device. Viscoelastography (Timanin, 2007; Rukavishnikov et al., 2017), tensomyography (García-García et al., 2019), and myotonometry (Kato et al., 2004; Lee et al., 2015; Arima, 2017; Nistorescu et al., 2019) methods are based on this principle.

Despite a wide variety of such methods, there is still a need for a highly accurate, portable, and non-invasive method. The availability of such a method would allow to reliably and accurately assess changes in muscle tone and consequently determine the degree of atony or atrophy. The myotonometry technique has the potential to become such a method. The principle of operation of MyotonPRO is that the device applies a mechanical impulse of stable strength and duration to the tissue. The tissue responds with damped oscillation and reflects the oscillation wave back to the device probe, where the oscillation is registered with an accelerometer in a form of acceleration signal. Based on this, frequency (Hz), stiffness (N/m), and other parameters are calculated (Schneider et al., 2015). The frequency directly depends on the intrinsic tension of the measured tissue and is considered to reflect muscle tone. Thus, the frequency value quantitatively characterizes the properties of the measured tissue and allows to assess the properties of individual muscles, including postural ones which are of particular interest for gravitational physiology.

Previously published studies have demonstrated high reliability of the MyotonPRO device in assessing assessing the mechanical properties of muscles both in the healthy population (Aird et al., 2012; Bailey, 2013) and in patients with pathological conditions (Lo et al., 2017; Van Deun et al., 2018). Furthermore, an experiment performed under microgravity conditions has confirmed that the MyotonPRO is an accurate, non-invasive, and easy-to-use device for assessing muscle tone as well as biomechanical and viscoelastic properties of muscles (Holt et al., 2016). However, the use of the handheld device can be affected by many factors, such as operator’s experience (Kim and Kim, 2016), assessment technique (Feng et al., 2018), and even background noise from the clinical environment (Van Deun et al., 2018). Therefore, the absence of a strict standardized research protocol makes it difficult to establish a range of standard values.

This problem is especially critical in gravitational physiology, where the levels of recorded changes depend on the data used as reference values. In space flight, the gravitational force is absent. Therefore, when orbiting the Earth, an astronaut can be correctly measured at any orientation of the body relative to the space module. However, at full rest, the muscle length and measurement point are required to remain close to the reference measurements obtained during the pre-flight measurement sessions.

Another problem is the insufficient knowledge about a sharp change in passive muscle tone. Despite the lack of direct evidence that the degree of atony correlates with the degree of atrophic changes, it is nevertheless well proven that atony precedes atrophy, triggering a cascade of cellular reactions (Shenkman and Kozlovskaya, 2019; Popova et al., 2020). A rapid drop in muscle tone in the first hours of weightlessness (or supportlessness) is likely to be a predictor of further disorders. A correct quantitative assessment of its decrease will help to adjust the system of assistance to astronauts, as well as to determine in the early stages the predisposition to back pain, proprioceptive illusions, severe atony e.c.t. and correct them.

The aim of our study was (i) to determine the initial effect of support presence/absence on muscle tone at the baseline, (ii) to determine the effect of 2-h supportlessness (Head-Up Dry Immersion), and (iii) to determine the changes in muscle tone depending on the measurement site. The authors also suggest a protocol for measuring muscle tone based on the data obtained that may be useful for future research in the field of gravitational physiology.

The null hypothesis of the study was the absence of significant differences in tone characteristics of the muscles under study (i) between normal support and supportlessness, (ii) between the very beginning of supportlessness and 2 h after the start of DI, (iii) between different measurement sites in the same muscle.

## Materials and Methods

### Study Population

The study involved 12 healthy volunteers without musculoskeletal disorders, 6 men and 6 women aged 24 to 42 years, with the average age of 32.8 ± 1.6 years (hereinafter, Mean ± SEM). The subjects were selected from the Russian population and had a normal level of physical activity. The subjects’ group had a normal distribution of body mass, height, body mass index. More details on the subjects’ data are given in Table 1.

**TABLE 1:** Subjects’ body parameters.

**Number of**	**Age, years**	**Height, M**	**Body mass,**	**Body mass**
**subjects, sex**			**Kg**	**index, Kg/M^2^**
1, Male	30	1.75	73.3	23.93
2, Male	33	1.59	62.4	24.68
3, Male	36	1.74	68.0	22.46
4, Male	42	1.77	87.0	27.83
5, Male	34	1.70	73.2	25.27
6, Male	38	1.78	80.1	25.39
7, Female	34	1.78	64.5	20.33
8, Female	39	1.55	46.8	19.61
9, Female	26	1.64	58.5	21.75
10, Female	28	1.72	68.3	23.11
11, Female	24	1.62	64.7	24.78
12, Female	30	1.68	69.5	24.77
Mean	32.8	1.69	68.0	23.66
SEM	1.6	0.02	3.0	0.67
SD	5.4	0.08	10.3	2.33
Shapiro-Wilk Test, *P*-value	*P* = 0.9880	*P* = 0.3195	*P* = 0.9393	*P* = 0.7653
Which Distribution	Normal	Normal	Normal	Normal
is more likely?	(52.33%)	(53.69%)	(59.3%)	(54.27%)

### Study Procedure

The experiment was performed using the DI bath; a more detailed description of the procedure can be found in previously published articles (Shul’zhenko and Will-Williams, 1976; Tomilovskaya et al., 2019). The study was carried out according to the protocols for 3- and 5-day Head Up Dry Immersion. Measurements were taken on the 1st - 4th days before DI on-couch and in-bath (baseline), and 2 h after the start of DI. No other examinations were performed during the first 2 h of DI. The subjects’ regimens during this period were not regulated and they had the possibility to relax or sleep.

Muscle tone was measured with a MyotonPRO device (Myoton AS, Estonia). All measurements were performed in the first half of the day by one operator. Any physical exercises before the experiment were prohibited. The measurement sites were determined by visual examination and palpation. The measurement points were marked on the skin over the most convex area known as the muscle belly. The marking was maintained throughout the study. The assessed trunk muscles included muscles (mm.) deltoideus posterior, trapezius, and erector spinae (in projections of T9-T8, T12-L1, L3-L4 segments of the spinal column). The leg muscles and tendons under study were: mm. biceps femoris, rectus femoris, tibialis anterior, soleus (lateral and medial part, plus central part over tendo Achillis), gastrocnemius (lateral and medial head, central between the heads). Two structures of the feet were measured – aponeurosis plantaris (between the first and second toes) and muscle (m.) flexor digitorum brevis (in the center of the feet). Also, the tone of tendo Achillis was measured.

**FIGURE 1:**
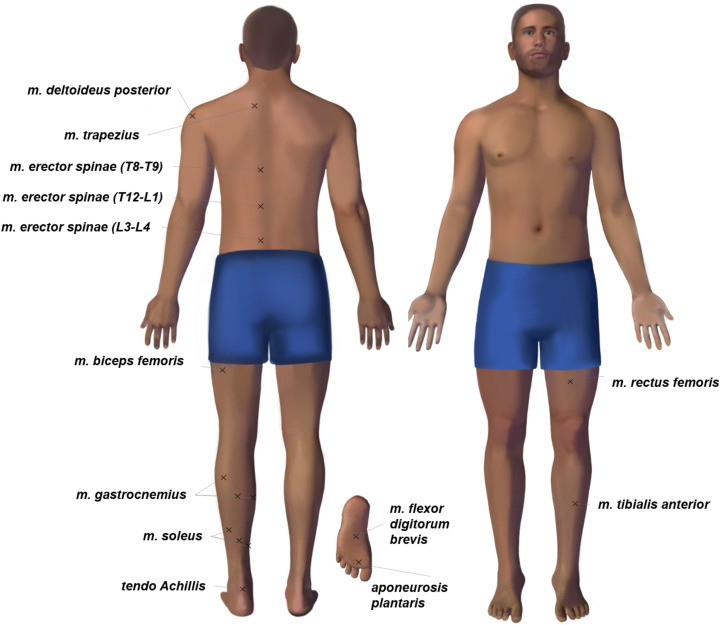
The body chart of tone measurement points. Each session of myotonometric measurements on premarked skin always started with mm. tibialis anterior, rectus femoris in the supine position and continued with m. flexor digitorum brevis, aponeurosis plantaris, tendo Achillis, soleus (three points), gastrocnemius (three points), biceps femoris, erector spinae, trapezius, deltoideus posterior in the prone position.

Note that only the muscles on the left side of the body were examined. A full list is given in Figure 1.

#### Subject’s Position

**FIGURE 2:**
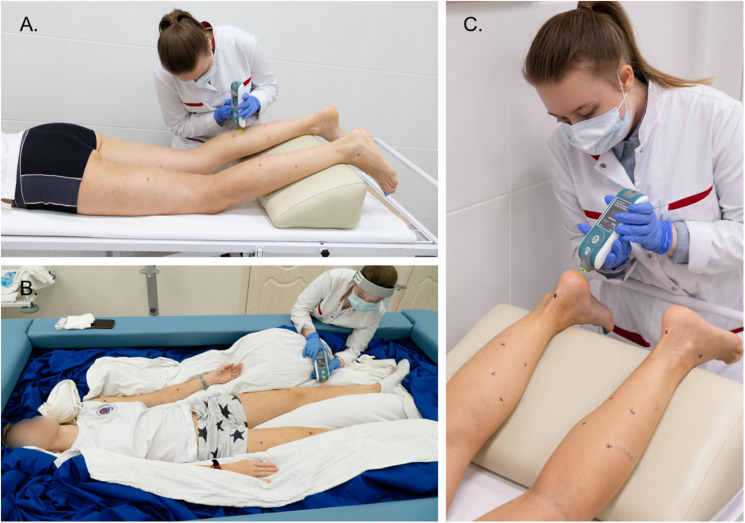
Examples of using the MyotonPRO device when measuring muscle tone of the leg **(A,B)** and foot **(C)**. The operator applies the device perpendicular to the muscles being tested. Tone measurement sites are indicated by black marks. Photos were taken and provided by the press secretary of the IBMP Oleg Voloshin.

**FIGURE 3:**
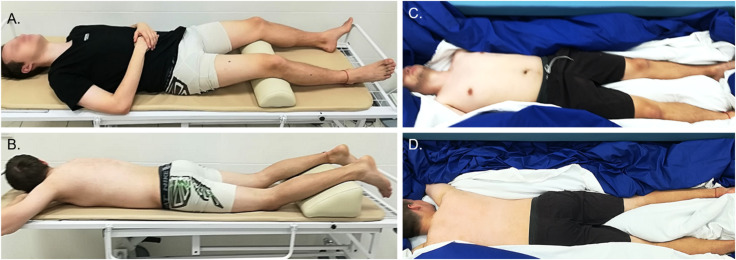
The subject’s position during on-couch **(A,B)** and in-bath **(C,D)** myotonometric sessions.

During the on-couch assessment, the subjects were in a supine and horizontal position with their hands placed in front of their heads. During the tone measurement procedure, all subjects were in a relaxed and immobile state. To standardize the angle of the leg joints, soft foam rubber for knees and ankles was used, as shown in Figures 2A,C, 3A,B.

For in-bath measurements, the subjects were laid on the waterproof film surface in supine (Figures 2B, 3C) or prone positions (Figure 3D). The subjects were instructed to be at full rest without any central nervous system activity while measurements were taken in a state of supportlessness. The foam rubber was not used because the film surface was comfortable enough.

#### Measuring Principle and Dependent Variables

A MyotonPRO wireless hand-held digital palpation device was used for measuring tone and biomechanical properties. The device probe was placed at the measurement points and held in the measurement position perpendicular to the skin surface (Figure 2). The green light of the device indicates the start of a fully automated measurement sequence which consists of a fixed preload (0.18N) to pre-compress subcutaneous tissues, a mechanical impulse (duration 15 ms, force 0.4N), and registering the tissue’s response to the impulse.

The tissue responds with damped oscillations and reflects the oscillation wave back to the device probe, where the oscillations are registered with an accelerometer as a form acceleration signal; the parameters are simultaneously computed and displayed on the device screen.

In this study, we analyzed only the frequency [Hz] parameter. The frequency characterizes the natural oscillations of the tissue in response to a mechanical impulse. The frequency parameter measured at full rest corresponds to a passive muscle tone which is an intrinsic state of tension at the cellular level (silent EMG signal), without voluntary contraction or CNS activity (Vain et al., 2015). The higher the frequency value the higher the tone or state of intrinsic tension of the muscles (Schneider et al., 2015).

### Statistical Analysis

The anthropometric parameters of normality of the subjects were checked using the Shapiro-Wilk test. The group was homogeneous in in terms of age, weight, height, and body mass index; all of these parameters had a normal distribution (Table 1). The data set for each muscle obtained using the same test also showed a normal distribution, which allowed us to apply parametric criteria for statistical analysis. A one-way repeated measures ANOVA with the Tukey’s *post hoc* test was used for muscle tone analysis, when comparing frequency changes under three conditions (on-couch baseline, in-bath baseline, and 2 h after the start of DI). To investigate the effect of measurement site on changes in muscle tone, a two-way ANOVA (time × measurement site) with the Tukey’s *post hoc* test was carried out. The significance criterion was set as <0.05.

## Results

### Changes in Tone of Trunk Muscles

**FIGURE 4:**
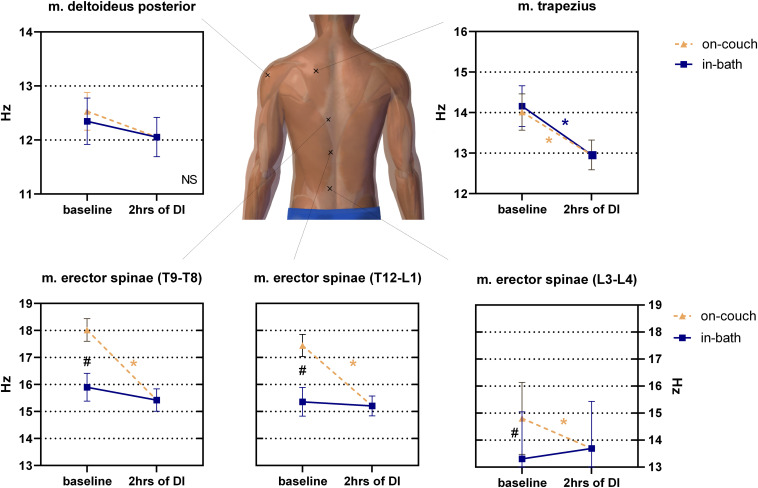
Changes in the tone (frequency) of the back muscles before and after 2 h of DI. Mean ± SEM. *P > 0.05 vs. baseline. ^#^*p* > 0.05 vs. on-couch.

No significant difference was found between on-couch (12.5 ± 0.4 Hz) and in-bath (12.3 ± 0.4 Hz) positions in m. deltoideus before DI (Figure 4A). The decrease in muscle tone was also insignificant after 2 h of DI (12.1 ± 0.4 Hz).

The initial baseline tone of m. trapezius was not sensitive to a change in position (14.0 ± 0.5 Hz on-couch, 14.2 ± 0.5 Hz in-bath, Figure 4). However, after 2 h of DI, the tone of this muscle significantly decreased to 13.0 ± 0.4 Hz (*F*(2,22) = 11; *P* < 0.001).

The muscle tone of m. erector spinae depended on the measurement site (Figure 4). The lowest muscle tone was observed at the lower level of m. erector spinae (projection of the L3-L4 segment of the spinal column). A significant difference (*F*(2,22) = 16.01; *P* < 0.0001) was found between the values on-couch (14.8 ± 0.4 Hz), in-bath (13.3 ± 0.5 Hz) and 2 h after the start of DI (13.7 ± 0.3 Hz). The middle level of m. erector spinae (projection of the T12-L1 segment of the spinal column) demonstrated higher muscle tone in both baseline conditions than the lower level of the erector spinae, but also had a significant difference between on-couch (17.4 ± 0.4 Hz) and in-bath (15.4 ± 0.5 Hz) values. Muscle tone decreased to 15.2 ± 0.4 Hz 2 h after the start of DI and a significant decrease was found compared to on-couch baseline values (*F*(2,22) = 21.96; *P* < 0.0001). A similar pattern was observed at the upper level of m. erector spinae (projection of the T9-T8 segment of the spinal column): 18.0 ± 0.4 Hz on-couch baseline, 15.9 ± 0.5 Hz in-bath baseline and 15.4 ± 0.4 Hz 2 h after the start of DI (*F*(2,22) = 20.43; *P* < 0.0001).

### Changes in Tone of Leg Muscles

**FIGURE 5:**
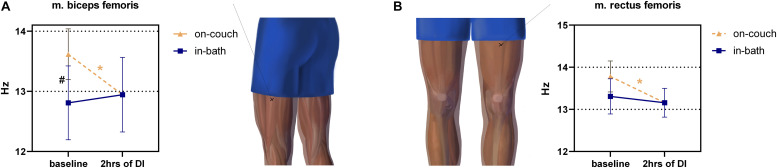
Changes in the tone (frequency) of the hip muscles before and after 2 h of DI. m. biceps femoris **(A)**, m. rectus femoris **(B)**. Mean ± SEM. *p > 0.05 vs. baseline. ^#^*p* > 0.05 vs. on-couch.

Significant changes were found in m. biceps femoris muscle tone between on-couch (13.6 ± 0.4 Hz) and in-bath (12.8 ± 0.5 Hz) baseline values (Figure 5A). The tone also significantly decreased (12.9 ± 0.6 Hz) after 2 h of DI (vs. on-couch, *F*(2,22) = 6.816; *P* = 0.005).

The tone of m. rectus femoris had a weak tendency to decrease (*F*(2,22) = 4,562; *P* = 0.0220) during DI, comprising 13.8 ± 0.4 Hz on-couch baseline, 13.3 ± 0.4 Hz in-bath baseline, and 13.2 ± 0.3 Hz after 2 h of DI (Figure 5B).

**FIGURE 6:**
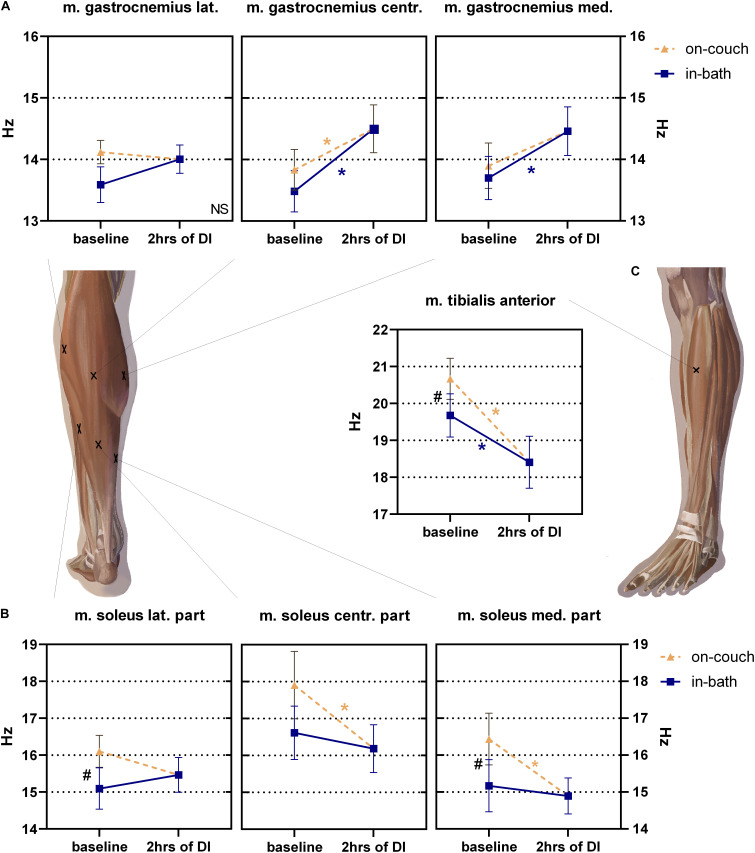
Changes of muscle tone (frequency) of mm. gastrocnemius **(A)**, soleus **(B)** and tibialis anterior **(C)** before and after 2 h into DI. Mean ± SEM. *P > 0.05 vs. baseline. ^#^*P* > 0.05 vs. on-couch.

Changes in the muscle tone of the shin varied in the degree and direction (Figure 6).

No significant changes were found in the tone of m. gastrocnemius lateralis; during the experiment, it varied from 13.6 ± 0.3 Hz to 14.1 ± 0.2 Hz (Figure 6A). The values of the tone of m. gastrocnemius centr. (between two muscle heads) were 13.8 ± 0.3 Hz on-couch and 13.5 ± 0.3 Hz in-bath, and significantly increased to 14.5 ± 0.4 Hz (*F*(2,22) = 10.63; *P* = 0.0006) 2 h after the start of DI. The baseline tone of m. gastrocnemius med. was 13.9 ± 0.4 Hz on-couch and 17.7 ± 0.4 Hz in-bath. After 2 h of DI, the tone increased by 14.5 ± 0.4 Hz and became significantly higher than in-bath baseline values (*F*(2,22) = 5.865; *P* = 0.0091).

The tone of the lateral part of m. soleus was significantly lower than on-couch (16.1 ± 0.4 Hz) and in-bath (15.1 ± 0.5 Hz, *F*(2,22) = 4.161; *P* = 0.0293) baseline values (Figure 6B). The tone values of m. soleus centr. were 17.9 ± 0.9 Hz on-couch and 16.6 ± 0.7 Hz in-bath. After 2 h of DI, muscle tone decreased to 16.2 ± 0.6 Hz (*F*(2,22) = 5.683; *P* = 0.0102). Similar changes were observed in medial part of m. soleus med: the tone values were 16.4 ± 0.7 Hz (on-couch baseline), 15.1 ± 0.7 Hz (in-bath baseline) and 14.9 ± 0.5 Hz (2 h after the start of DI) (*F*(2,22) = 9.780; *P* = 0.0009).

The muscle tone of m. tibialis anterior was 20.7 ± 0.5 Hz on-couch and 19.7 ± 0.6 Hz in-bath (Figure 6C). A 2 h after the start of DI, a decrease in tone to 18.4 ± 0.7 Hz was recorded; it was significantly different in all three sites (*F*(2,22) = 26.65; *P* < 0.0001).

### Changes in Tone of Foot Muscles

**FIGURE 7:**
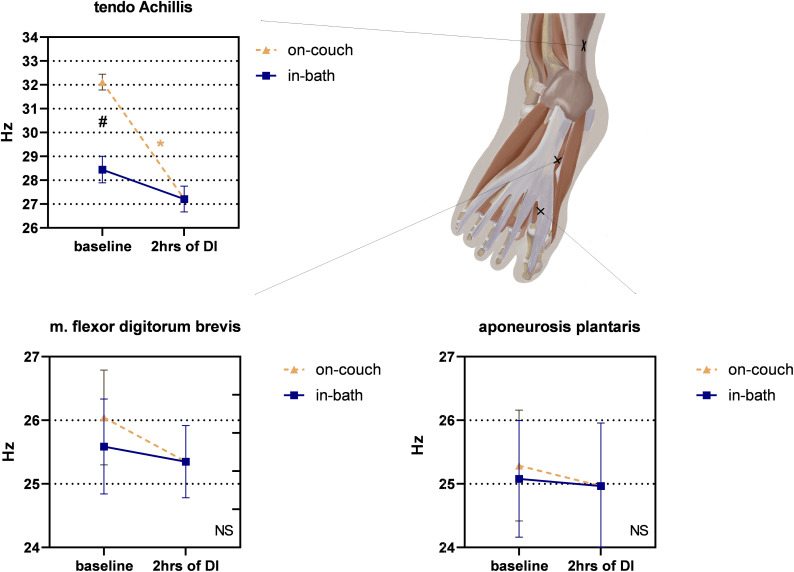
Changes in the structure of the feet and the tone of the tendo Achillis before and after 2 h of DI. Mean ± SEM. *P > 0.05 vs. baseline. ^#^*P* > 0.05 vs. on-couch.

The on-couch baseline tone of the tendo Achillis was the highest – 32.1 ± 0.3 Hz (Figure 7). The in-bath baseline tone was significantly lower – 28.4 ± 0.5 Hz. 2 h after the start of DI, a significant decrease in tone compared with on-couch baseline values was observed – 27.2 ± 0.5 Hz, *F*(2,22) = 45.82; *P* < 0.0001).

No significant changes were found in m. flexor digitorum brevis, though a downward trend was observed (Figure 7). The tone values were 26.0 ± 0.7 Hz on-couch, 25.6 ± 0.7 Hz in-bath, and 25.3 ± 0.6 Hz after 2 h of DI.

No significant changes were found in aponeurosis plantaris, however, a slight tendency to increase was noted (Figure 7). Both on-couch and in-bath baseline tone values were identical – 25 Hz; even after 2 h of DI, the tone remained at the same level (25.0 ± 1.0 Hz).

**FIGURE 8:**
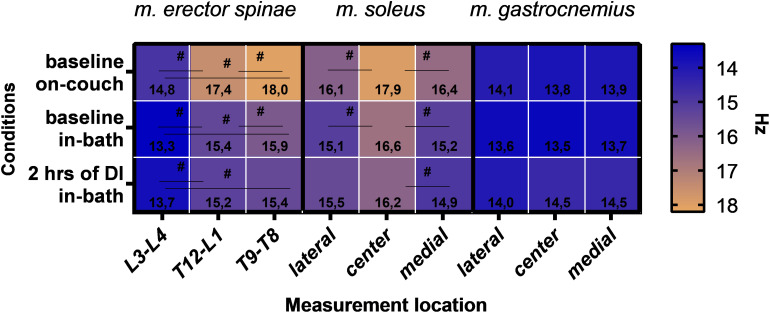
Muscle tone data for mm. erector spinae, soleus and gastrocnemius according to the site of measurement. Mean. ^#^*P* > 0.05.

A comparative analysis was carried out for the measurement sites of three muscles (mm. erector spinae, soleus, gastrocnemius). As shown in Figure 8, data of mm. erector spinae (*F*(2,22) = 34; *P* < 0.0001) and soleus (*F*(2,22) = 5.0; *P* = 0.0158) depend on the measurement site. No significant differences were found for the measurement sites in m. gastrocnemius.

In addition to the main results, percentage changes in muscle tone were also calculated (Table 2). We noticed that the baseline data obtained on-couch and in-bath can vary greatly. As mentioned above, many muscles show a significant decrease in muscle tone compared to on-couch data. It is important to take into account relative to which value the changes will be calculated during the experiment. In this regard, we calculated the percentage changes relative to three experimental conditions. The results are shown in Table 2.

**TABLE 2:**
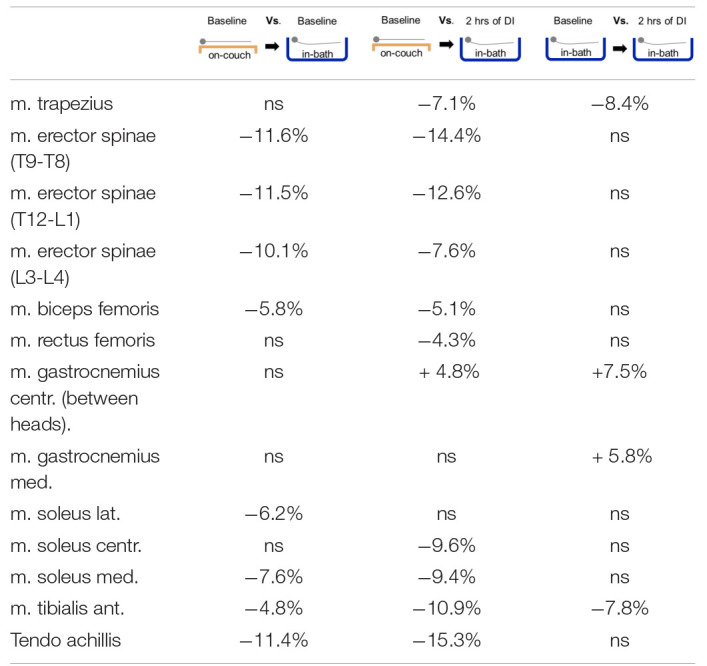
Percentage difference of muscle tone between three experimental conditions.

*Values are shown for significant changes only.*

## Discussion

The obtained results demonstrated: (i) a significant decrease in the tone of mm. erector spinae, biceps femoris, tibialis anterior, soleus muscle and associated tendo Achillis immediately after the transition to a state of supportlessness; (ii) the effect of 2-h supportlessness varied depending on what initial data were taken as reference values. If the on-couch data were taken as the baseline, then a significant decrease in the muscle tone of postural muscles (mm. erector spinae, soleus, rectus femoris, biceps femoris), mm. trapezius, tibialis anterior and tendo Achillis was observed after 2 h of DI. If the in-bath data were taken as reference values, then significant differences were found in fewer muscles; and (iii) furthermore, changes in muscle tone of mm. erector spinae and soleus were site-dependent.

Despite the fact that a decrease in muscle tone was shown at the dawn of manned astronautics (Cherepakhin et al., 1973), no exact values of the decrease in muscle tone have yet been established. This is likely due to the lack of a “gold standard” method and low scientific interest in muscle tone in favor of muscle strength research. The only published study of muscle tone under conditions as close as possible to real space flights (Schneider et al., 2015) shows a significant and immediate decrease in tone and stiffness of mm. erector spinae, gastrocnemius med. and tendo Achillis in a parabolic flight. However, due to the short (20 s) time of exposure to microgravity, it is not possible to draw conclusions about further changes in muscle tone.

In earlier studies (de Abreu et al., 2017; Rukavishnikov et al., 2017), a decrease in the tone of m. erector spinae under immersion conditions has been reported. It is interesting to note that the severity of changes of m. erector spinae depended on the measurement site – the upper parts of the muscle were more susceptible to the effects of supportlessness. At the same, it has previously been shown that the presence of functional muscular asymmetry does not significantly affect the degree of decrease in muscle tone. In general, it may be concluded that changes in the tone of the back muscles progressively increased in the direction of the sterno-lumbar spine. However, research results show that the m. erector spinae muscle is homogeneous in composition along its entire length and contains 60–70% of type I fibers (Mannion et al., 1997; Agten et al., 2020). Therefore, a smaller decrease in muscle tone in our study can not be explained by a lower content of tonic muscles in the lumbar region. We hypothesize that the nature of this phenomenon lies in functional changes during the transition to supportlessness conditions and may be associated with back pain that develops on the first day of immersion (Plehuna, 2015; Rukavishnikov et al., 2017). Thus, further in-depth research of back muscles is needed to investigate a large number of interrelated factors that could trigger back pain syndrome under weightlessness conditions.

The soleus muscle is known to be predominantly composed of slow fibers [70% (Edgerton et al., 1975)] which are directly involved in maintaining posture. In our study, the m. soleus is one of the muscles of the posture group and is sensitive to gravitational unloading; the observed changes are in full agreement with previous experimental results (Kozlovskaya et al., 1988, 2007; Shenkman and Kozlovskaya, 2019). However, the most significant changes were observed in the central projection of the muscle covered with the tissues of tendo Achillis which is a rigid structure. The current study showed that the baseline tone of the soleus medial and lateral parts were lower than that of the central part. After 2 h of immersion, muscle tone decreased by 9.6% compared to the on-couch data. It is most likely that the measurement of the soleus muscle tone in this projection is the most informative and reliable. In a study on the stiffness of tendo Achillis, Morgan et al. (2018) has shown that the data can vary by up to 30% in the prone position and by 50% in the upright position depending on the measurement site (distal or proximal). It is interesting that the tendo Achillis which is the most rigid structure among the studied showed a significant decrease in its tone immediately after beginning of DI and even further relaxation with a longer exposure to DI. This observation is similar to the data from the study by Schneider et al. (2015), where exposure to microgravity during repetitive parabolic flights has led to a rapid relaxation of the tendo Achillis. The study has shown that changes in the tendo Achillis may occur due to a general body relaxation that should be easier to detect with more rigid tissues such as tendons (Schneider et al., 2015).

Assessment of the gastrocnemius muscle showed an increase in tone after exposure to DI that was not previously recorded. This reaction is likely to be associated with fascial contractility (Schleip et al., 2019) or pennate muscle changes (Hullfish et al., 2019). We hypothesize that the fascia may compensatorily contract in response to an abrupt decrease in muscle tone. Undoubtedly, this assumption requires further research and verification.

The present research also had some limitations. The experiment was based on the protocol of Schoenrock et al. (2018). The muscles were examined only on the left side of the body, as the aim of the present work did not include any asymmetry studies. However, this allowed us to investigate more muscles, which is an advantage. Expanding the list of studied muscles allows to get a more comprehensive picture of changes in muscle characteristics. It is crucial to study the tone of the back and lower leg muscles in more detail, since postural stability depends on the function of these muscles. Even though we did not measure the muscle tone in standing position, it is obvious that the obtained data would be useful for studies in the field of gravitational physiology. Further studies will focus on this direction, as the level of tonic activity is fundamentally different in lying and standing positions (Morgan et al., 2018). Moreover, there is a limitation of the myotonometer technique itself, since it measures not only the properties of a specific muscle structure, but can also be affected by the properties of the soft tissues above the muscle fibers. This issue has been studied in depth by other researches and data from various experiments have shown that muscle stiffness measured by myotonometer technique at rest moderately correlates with muscle stiffness measured by elastography (Hu et al., 2018; Kelly et al., 2018). It is important to note that the data from this study were obtained as part of much longer than 2 h protocols (3 and 5 days) aimed at solving other problems. These data will be published elsewhere.

The high sensitivity of the myotonometry technique prompts us to design a unified research protocol for measuring muscle tone in order to obtain comparable data. The authors propose to organize a conference and approve a unified research protocol for use in gravitational physiology.

## Conclusion

A decrease in postural muscle tone occurred immediately after the start of immersion and intensified after the first 2 h of immersion. The degree of muscle change depended on the experimental condition that was chosen as the reference value (on-couch or in-bath). Moreover, the measurement site was critical for assessing the degree of change in muscle tone within a single muscle.

To make it possible to compare data from different studies, a standardized protocol for measuring muscle tone for general use in the field of gravitational physiology needs to be developed.

## Data Availability Statement

The original contributions presented in the study are included in the article/supplementary material, further inquiries can be directed to the corresponding author.

## Ethics Statement

All the subjects gave written informed consent according to principles of the Declaration of Helsinki and approvedprocedures of the Bioethics Committee of the Institute of Biomedical Problems of the Russian Academy of Science (protocols # 465 of 25.12.2017 and # 544 of 16.07.2020). The subjects signed informed consent for posting of identifying information or images in an open-access online publication.

## Author Contributions

LA designed the study, collected the data, and wrote the draft of the manuscript. APl made a major revision of the manuscript and proofreading. IR and AS helped with the study design, organized medical support, and contributed to the revision of the manuscript. APe contributed to creation of the study design and made a revision of the manuscript. ET was the supervisor of the study and contributed to the revision of the manuscript. All authors contributed to the article and approved the submitted version.

## Conflict of Interest

APe was employed by Company Myoton AS, Estonia. The remaining authors declare that the research was conducted in the absence of any commercial or financial relationships that could be construed as a potential conflict of interest.
